# Resolving the clinico-radiological paradox in multiple sclerosis

**DOI:** 10.12688/f1000research.11932.1

**Published:** 2017-10-12

**Authors:** Declan Chard, S Anand Trip

**Affiliations:** 1 National Institute for Health Research (NIHR) University College London Hospitals (UCLH), Biomedical Research Centre, London, UK; 2 NMR Research Unit, Queen Square Multiple Sclerosis Centre, UCL Institute of Neurology, London, UK

**Keywords:** multiple sclerosis, clinico-radiological paradox, MRI

## Abstract

Understanding the clinico-radiological paradox is important in the search for more sensitive and specific surrogates of relapses and disability progression (such that they can be used to inform treatment choices in individual people with multiple sclerosis) and to gain a better understanding of the pathophysiological basis of disability in multiple sclerosis (to identify and assess key therapeutic targets). In this brief review, we will consider themes and issues underlying the clinico-radiological paradox and recent advances in its resolution.

## Introduction

The clinical course of multiple sclerosis (MS) is diverse and unpredictable: Some people develop rapidly evolving neurological deficits, whereas others accrue little or no detectable neurological deficit over decades ^1^. Magnetic resonance imaging (MRI) is now firmly established as a key tool in the diagnosis of MS ^2^, based on its ability to show white matter (WM) lesions with a typical spatial and temporal distribution for MS. However, the number and volume of WM lesions explain only a small fraction of the diversity of clinical outcomes in MS, and this mismatch has been termed the clinico-radiological paradox ^3^.

This apparent paradox would matter less if we had clinical tools that were able to sensitively and reliably measure MS progression; however, scores such as the Expanded Disability Status Scale (EDSS) have well-recognised limitations ^4^, one of which is relatively limited sensitivity to change in individuals. Some MRI measures are much more sensitive to change, but their clinical relevance is debated, in part due to the clinico-radiological paradox. Despite this, and because of greater sensitivity to change, MRI is now routinely used as the primary outcome in early phase treatment trials. While associations between the accrual of WM lesions and MS relapses are modest, it has been shown that a treatment effect seen on WM lesion accrual is a good predictor of the likely effects on relapses ^5^.

The clinico-radiological paradox has also assumed a greater relevance with the advent of treatments that suppress relapses and, very recently, slow progression. Given the limited sensitivity of clinical measures for ongoing disease activity, there has been increasing interest in using composite clinical and MRI measure of disease activity and treatment efficacy (for example, ‘no evidence of disease activity’ assessments based on relapses, clinical progression and MRI measures of new lesion formation). However, in this regard, low levels of MRI-apparent disease activity may not (on their own) be associated with a substantial risk of further clinical events ^6^.

Understanding the clinico-radiological paradox is important in the search for more sensitive and specific surrogates of relapses and disability progression (such that they can be used to inform treatment choices in individual people with MS) and to gain a better understanding of the pathophysiological basis of disability in MS (to identify and assess key therapeutic targets). In this brief review, we will consider themes and issues underlying the clinico-radiological paradox and recent advances in its resolution.

## Pathological abnormalities in multiple sclerosis

The pathological effects of MS are multifaceted and variable, and the location, nature, extent and intensity of pathology are all relevant to clinical outcomes. While the most conspicuous abnormality is the presence of demyelinating lesions in WM, there is much more to the pathology of MS than WM lesions. Grey matter (GM) lesions are seen throughout the course of MS ^7^ and, in people with long-standing progressive MS, may actually be more extensive than lesions in WM ^8^. Extra-lesional tissues are also abnormal with, for example, glial abnormalities and reduced axonal densities seen in WM ^9^ and glial abnormalities, and loss of dendrites and neurons, in GM (for example, 10). Lesions themselves are heterogeneous, sufficiently so that WM subtypes have been defined ^11^, and within lesions there may be differing degrees of inflammation, demyelination and remyelination, and axonal loss, all of which may influence their functional consequences. Pathology in MS is also not spatially uniform. Demyelinating lesions tend to form around the lateral ventricles ^12^, and GM lesions occur most often in the cortical layers close to the surface of the brain ^8^. Neurodegeneration also affects some regions more than others (for example, retrograde neurodegeneration may preferentially affect deep GM) ^13^. Elements of MS pathology (for example, brain WM lesion loads) can vary substantially among people with MS, even when they have seemingly similar clinical outcomes.

## Measurement characteristics

**Figure 1.**
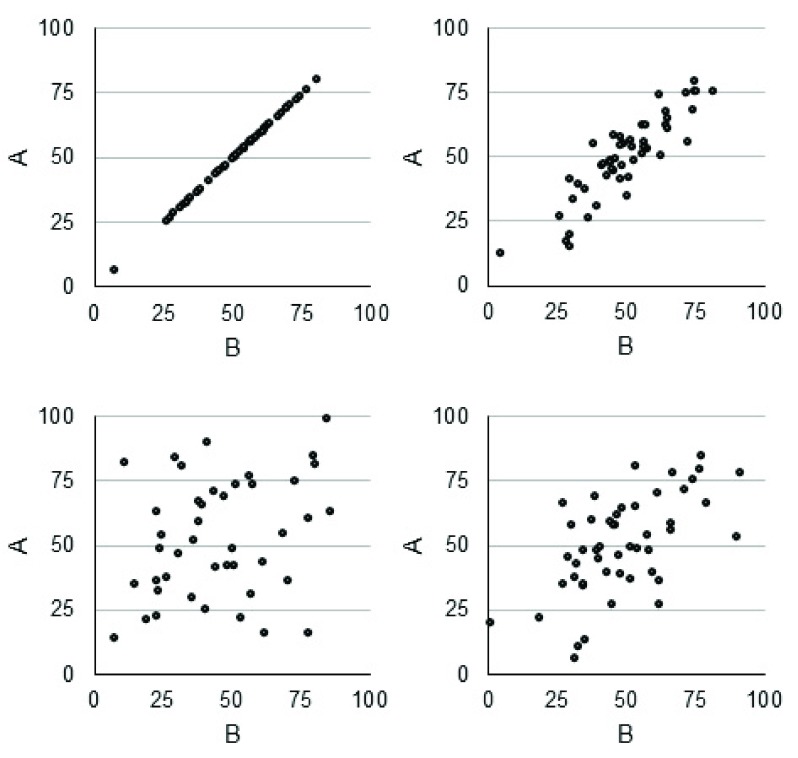
Simulated perfectly correlated data, with a mean of 51 and a standard deviation (SD) of 15.1. Clockwise from the top left: No added noise, r = 1; noise SD = 5, r = 0.89; noise SD = 7.5, r = 0.59; and noise SD = 10, r = 0.33.

When we measure, we do so with varying degrees of overlying noise and underlying specificity, both of which can significantly reduce the apparent strength of associations between measures. When we repeat a measure, it is usual to observe variations in the results; whereas some of this variation may be due to a change in what is being measured, some will be due to measurement error; and with increasing measurement errors, apparent associations between measures can be attenuated ( Figure 1). This can lead us to underestimate the clinical relevance of a pathological feature being assessed by using MRI. Similarly, the specificity of a measure can significantly affect associations: Few (if any) MRI or clinical measures reflect only one biological feature or its associated functional outcome, although some are more specific than others (see below).

## Clinical measures

Clinical measures in MS seek to assess either the frequency of events over a given period (for example, relapse rate) or the level of neurological impairment at a given time. The most frequently assessed clinical measure of disease activity is the relapse rate. However, this has a number of limitations. It reflects new inflammatory lesions in clinically eloquent regions only, and lesions in other areas will go undetected. The estimation of a relapse rate is also open to recall bias (particularly where events are not recorded at the time of their occurrence), inter-observer variability (as each clinician may apply different clinical thresholds), and other factors such as silent infection, fatigue and low mood which can give rise to pseudo-relapses (that is, a transient worsening of symptoms that is not due to new inflammatory activity) ^14^.

With regard to measures of neurological impairment, the EDSS ^15^ is the most widely used measure of disability in MS. It produces a score based on symptoms and signs in eight functional systems, walking ability and activities of daily living. Scores are on an ordinal scale from 0 (asymptomatic with normal examination) to 10 (death due to MS). Scores of 0–3.5 reflect neurological impairment without a clear effect on mobility, 4–7.5 reflects impaired mobility, and 8–9.5 reflects effects on activities of daily living. Though easy to record and widely used, it has several limitations. For an ordinal scale measurement, parametric statistics should not be used without assessing whether the underlying assumptions of such statistics have been significantly breached, and non-parametric statistics are often preferred albeit with a potential loss of statistical power. It is also open to intra- and inter-variability when scoring, particularly with scores of less than 4 ^16^. Since its initial description, it has become apparent that other frequently occurring—and functionally significant—clinical manifestations of MS, such as cognitive impairment and fatigue, are relatively overlooked, and therefore it is not a true global measure of disability ^4^.

To address some of the limitations of the EDSS, the Multiple Sclerosis Functional Composite (MSFC) was developed ^17^. It assesses three important functional domains: ambulation (timed 25-foot walk), upper limb function (nine-hole peg test) and cognition (paced auditory serial addition task, or PASAT) ^17^. The results from each component are converted to a z-score based on values from a reference population, thus producing continuous rather than ordinal data. The MSFC has good intra- and inter-rater reliability and, importantly, also more explicitly assesses cognition. However, there is debate about whether to use the MSFC score or its components separately. Combining domains provides a relative assessment of the clinical effects of MS; but, unlike the EDSS score, it does not provide an easily interpretable measure of disability; for example, an EDSS score of 3.5 means that mobility is not impaired, whereas an MSFC z-score of 3.5 does not do so. In addition, small but significant changes in any of the domains may be diluted and so overlooked. Given that each domain included in the MSFC may be influenced to a greater or lesser degree by different aspects of MS pathology (for example, cortical neurodegeneration may be more relevant to cognitive impairment than motor function, whereas demyelination or axonal loss may be more relevant to motor function than cognitive impairment), the use of more specific clinical outcome measures is likely to be more informative when investigating pathophysiological mechanisms.

While the MSFC is more comprehensive in its assessment of disability than the EDSS, there is still scope for this to be improved on. The PASAT has some practical drawbacks such as a requirement for mathematical skills, a ceiling effect, and it is disliked by test subjects. Furthermore, it does not encompass all aspects of cognitive function. Other measures of cognitive dysfunction have been proposed. The Symbol Digit Modalities Test (SDMT) measures information-processing speed and is more easily administered, better tolerated, and more reliable than the PASAT ^18^. Building on the SDMT, a brief international cognitive assessment for MS (BICAMS) has been proposed which includes the SDMT with the addition of the California Verbal Learning Test and the Brief Visuospatial Memory Test ^19^. This attempts to assess other aspects of cognitive dysfunction. The MSFC also does not assess domains such as vision and bladder or bowel function, which can also have substantial effects on the lives of people with MS ^4^.

While the EDSS is not an ideal measure of the neurological effects of MS, it remains the most popular disability score in clinical practice, and pragmatically (owing to service constraints) it would be difficult to incorporate a more extensive assessment in most healthcare settings. In clinical trials, while there may be more scope to use additional clinical outcome measures, for new treatments to be licensed these would have to be accepted by regulatory authorities as being valid markers of efficacy.

## Magnetic resonance imaging measures

MRI measures can be broadly divided into those that assess macro-structure (for example, brain volumes), micro-structure (for example, magnetisation transfer imaging and diffusion tensor imaging, or DTI), metabolic features (such as proton spectroscopy and perfusion) and function (functional MRI, or fMRI) ^20^. Here, to illustrate points, we will consider these in outline only.

As with pathological studies, WM lesions in MS are the most conspicuous abnormality on conventional MRI scans. The majority of WM lesions seen on histopathological examination are also identified on MRI, and about 60% are seen on T2-weighted scans and 70% on T2 fluid-attenuated inversion recovery (FLAIR) scans ^21^. Though more extensive, GM lesions are much more difficult to identify on these scans; less than 5% of cortical lesions are seen on T2-weighted scans, and 5% on T2-FLAIR; for deep GM, about 15% and 40% respectively are seen ^21^. Double inversion recovery improves on this; about 10% of cortical and deep GM lesions are detected ^22^, and phase-sensitive inversion recovery may increase this further ^23^. However, while subpial GM lesions are the most extensive type seen histopathologically, they are rarely identified using MRI at 3T. MRI at higher field strengths, such as 7T, appears to improve on this ^24^ but currently is not widely available in clinical practice or for research. Seewann and colleagues have shown (at 1.5T) that the GM lesions detected using MRI are the ‘tip of the pathological iceberg’, differing in size rather than other pathological characteristics from those that go undetected ^25^. Practically, when correlations of lesions measures with clinical outcomes are considered, it is likely—given lower sensitivity to GM compared with WM lesion—that the clinical relevance of GM lesions (in particular subpial lesions) will be underestimated when compared with WM lesions.

It is well recognised that lesions may be pathologically diverse but can appear similar on MRI scans. For example, without the use of a contrast agent, it is not possible to determine whether a lesion is actively inflamed and demyelinating, chronically demyelinated or remyelinating. However, not all lesions seen on a T2/proton density-weighted scan are seen on a T1-weighted image, and the subset of lesions seen on T1-weighted scans appear to represent those that have sustained greater axonal injuries ^26^. Despite this, correlations of T2-weighted and T1-weighted WM lesion loads with clinical outcomes are similar ^27^, suggesting that the difference in axonal loss between these lesions is either of limited clinical relevance or that other factors (such as measurement errors) mask any additional contribution axonal loss may make (beyond demyelination) to the association between lesion measures and clinical outcomes.

Brain volume loss, a marker of brain atrophy, which reflects neurodegeneration ^28, 29^, is an MRI measure that is increasingly being used in clinical trials, particularly in progressive MS. However, the optimal way to measure brain volume or atrophy has yet to be determined, and a variety of approaches have been tried ^30^. Methods can be divided into those that measure tissue volumes on each MRI scan independently and those that compare scans to assess differences. At a whole brain level, methods that compare scans (for example, as implemented in Structural Imaging Evaluation, using Normalization, of Atrophy, or SIENA) rather than assess each scan independently appear to be significantly more robust and sensitive to differences ^31^. However, brain volume loss does not affect all brain tissues and regions equally; for example, there is evidence that it occurs more rapidly in GM than in WM ^32^. As such, regional brain volume loss may be diluted in whole brain measures, and it has proven challenging to develop regional volume measures that can robustly compare scans.

So far, we have considered MRI measures of the quantity of a given tissue, but, as noted above, clinically relevant pathological abnormalities can affect the quality of tissues too. For example, in normal-appearing WM, magnetisation transfer ratio (MTR) and DTI measures both have been shown to correlate with disability measures ^33^. However, measures of intrinsic tissue characteristics are not pathologically specific. For example, MTR correlates not only with myelin density but also with axonal density (which itself is correlated with myelin density) and gliosis ^34, 35^. In progressive MS, neurodegeneration is thought to be a (if not *the*) major process responsible for neurological deficits ^36^. While brain atrophy reflects neurodegeneration, loss of axons and neurons does not necessarily lead to an equivalent reduction in brain volumes: With an accurate measure of neuronal and axonal density, we would be able to better estimate the actual amount of neurodegeneration rather than a small fraction of it.

Most MRI measures used in MS research assess structure, but some investigate metabolic and (indirectly) neuronal function. Of these, fMRI has been most studied to date but highlights the challenges of trying to assess localised neural activity ^37^. Neuronal activity occurs at the level of single cells, but fMRI works at a much coarser scale (typically several cubic millimetres). fMRI also does not assess neuronal activity directly but instead via its effects on blood flow and oxygen extraction. Therefore, there is a delay between neuronal activity and what is measured by fMRI. Other factors (both physiological and pathological) can significantly affect blood flow and oxygen extraction and so can further decouple fMRI associations with underlying neuronal activity. There are two main forms of fMRI: task-based (with a subject performing a specific activity, such as finger tapping or counting) and resting-state (with a subject at rest). These provide different information; task-based fMRI probes specific networks (for example, motor), whereas resting-state offers a more global but less network-specific assessment of brain function ^37^. Interpretation of fMRI findings is challenging, as there may be decreased and, paradoxically, increased regional activity in MS ^38^. Increased activation may be adaptive (reducing the functional impact of MS pathology) or maladaptive (having no beneficial effect on function or perhaps even being associated with greater functional impairments) ^39^, but these cannot be readily distinguished in cross-sectional studies. To determine the dynamic relationship between fMRI measures and clinical outcomes, longitudinal studies are required to clarify this ^39^.

## Linking pathology to clinical outcomes

**Figure 2.**
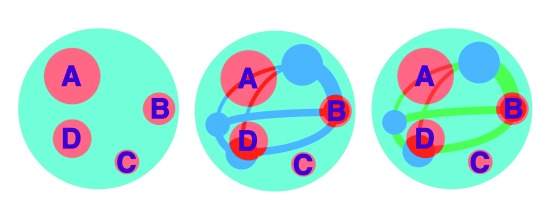
Schematic of the brain (light blue), a brain network (dark blue and green) and lesions (red). From left to right: Whole brain lesion measures; a region of interest-based assessment of lesions within the network of interest; a network-based assessment of lesions. In the whole brain assessment (left), lesion C will have a negligible effect on network performance and so will dilute associations with the associated clinical outcome. In a region of interest-based assessment of the network (middle), the effects of lesions A, B and D are considered proportional to the volume of the network involved and so may relatively over- or under-value the effects that each lesion has on the associated clinical outcome. In the network-based assessment (right), lesions in each brain region (dark blue) or connecting tract (green) are considered separately, so allowing the effects on outcomes to be weighted according to their location within the network. For example, lesion B involves three tracts and so may have a greater effect on network performance than lesions A and D (each of which affects two tracts).

So far, we have noted technical issues with, and the composite nature of, clinical and MRI measures that may attenuate correlations. We have also considered how MS disease effects are not evenly distributed and that regional effects may be diluted in whole brain measures and so overlooked. However, we have yet to consider the way in which the central nervous system is organised and in particular how neurological functions rely on the integrity of neural networks. This has significant implications for the way in which we seek to connect MS pathology with clinical outcomes, as pathology within networks may be undervalued in whole brain measures ( Figure 2). Recognising this, studies have looked for associations between regional pathology and clinical outcomes. For example, with motor outcomes, it has been shown that abnormalities in the cortico-spinal tract are more closely linked with function than are measures derived from elsewhere in the brain ^40^. Similarly, when compared with whole brain measures, temporal lobe atrophy has been more closely linked with memory impairments ^41^, as has more specific hippocampal atrophy ^42^. Another region of particular interest in MS is the thalamus, which may be affected directly and through its extensive connections with other brain regions ^43^. It has been suggested that, owing to its highly connected nature, the thalamus may act as a ‘barometer’ of MS pathology ^43^. Although we have focused on brain measures in this review, it is also clear that pathology anywhere in the central nervous system can affect clinical outcomes in MS and may do so in addition to abnormalities in the brain (for example, spinal cord atrophy ^44^).

Furthermore, connectomics studies have started to model the integrity of networks from MRI data (that is, the ability for neural networks to transfer and process information and so deliver a functional outcome) ^45^. Rather than treating a network as a region and using MRI to measure pathological effects averaged across it, investigators assess elements of the network separately and treat them as components, each of which has a different contribution to functional outcomes. Graph theory ^45^ provides a mathematical framework with which to model overall network characteristics on the basis of brain regions and the connection between them. A variety of measures can be derived from a graph theory analysis, and it has yet to be determined which measure (or measures) best represents the effects that pathology has on function. Despite this uncertainly and complexity, such analyses have already shown some promise in MS. For example, correlations of EDSS with network efficiency (calculated using DTI measures from motor network tracts, Spearman r = −0.52) are noticeably greater than correlations of EDSS with conventional MRI measures (log T2 lesion load r = 0.27 or brain volumes r = −0.30) ^46^. Associations of network efficiency with EDSS appear to increase further when two measures of tissue microstructure (DTI and MTR) are combined (r = −0.77).

In addition to structural MRI measures in MS, graph theory has been applied to functional measures such as fMRI (for example, disruption of network integrity has been linked with cognitive impairment ^47, 48^) and magnetoencephalography data (showing abnormalities when comparing people with MS and controls ^49^). Considering structural and functional assessments of the brain together can provide useful insights into the mechanisms underlying disability in MS (for example, ^38^), but combining structural and functional measures in a unified assessment of network performance has, to the best of our knowledge, not yet been achieved in MS.

## Conclusions

Recognition of the clinico-radiological paradox in MS has inspired considerable research and has led to a reconsideration of MRI and clinical measures and the link between them. Since this was first raised, there has been considerable progress on several fronts, to a degree that the mismatch between clinical and MRI measures is less an unexplained paradox (and so perhaps the term ‘clinico-radiological paradox’ is increasingly a misnomer) and more a potentially reconcilable challenge. From an MRI perspective, there is growing interest in regional and network-specific assessments of pathology and in developing more pathologically specific measures. From a clinical outcomes perspective, composite scores (such as EDSS) are being rethought, and measures of more specific functions developed. Linking MRI measures and clinical outcomes remains challenging, but insights from connectomics studies are helping to bridge the gap.
